# Cytoreductive Nephrectomy in Select Primary Metastatic Renal Cell Carcinoma Patients: A Comprehensive Nationwide Outcome Analysis

**DOI:** 10.3390/cancers16061132

**Published:** 2024-03-12

**Authors:** Nessn Azawi, Louise Geertsen, Naomi Nadler, Karina Sif Soendergaard Mosholt, Sofie Staal Axelsen, Jane Christensen, Niels Viggo Jensen, Niels Fristrup, Susanne Oksbjerg Dalton, Frede Donskov, Lars Lund

**Affiliations:** 1 Department of Urology, Zealand University Hospital, Sygehusvej 10, 4000 Roskilde, Denmark; 2 Institute for Clinical Medicine, University of Copenhagen, Noerregade 10, 1165 Copenhagen, Denmark; sanne@cancer.dk; 3 Department of Urology, Odense University Hospital, J. B. Winslows Vej 4, 5000 Odense, Denmarklars.lund@rsyd.dk (L.L.); 4 Institute of Clinical Medicine, University of Southern Denmark, Campusvej 55, 5230 Odense, Denmark; 5 Department of Urology, Rigs Hospital, Blegdamsvej 9, 2100 Copenhagen, Denmark; karina.sif.soendergaard.mosholt@regionh.dk; 6 Department of Oncology, Aarhus University Hospital, Palle Juul-Jensens Blvd. 161, 8200 Aarhus, Denmarkniels.fristrup@rm.dk (N.F.); 7 Danish Cancer Society, Strandboulevarden 49, 2100 Copenhagen, Denmark; 8 Department of Oncology, Odense University Hospital, J. B. Winsløws Vej 4, 5000 Odense, Denmark; niels.viggo.jensen@rsyd.dk; 9 Department of Clinical Oncology and Palliative Care, Zealand University Hospital, Radmandsengen 5, 4700 Naestved, Denmark; 10 Department of Oncology, Southern Denmark University Hospital, Esbjerg, Finsensgade 35, 6700 Esbjerg, Denmark; fdonskov@health.sdu.dk

**Keywords:** cytoreductive nephrectomy, real world data, renal cell carcinoma

## Abstract

**Simple Summary:**

This stduy focuses on a specific surgery for advanced kidney cancer that has spread, known as cytoreductive nephrectomy. The study examined the health results of 437 patients over five years to see if the surgery, when combined with drug therapy, offers better outcomes than just the drug therapy on its own. The findings suggest that patients who underwent both the surgery and the drug therapy tended to fare better than those who only had the drug therapy. This indicates that the surgery might be a good option for some patients, but more studies are needed to be sure. The results of this study could help doctors make better treatment plans for patients with this type of kidney cancer in the future.

**Abstract:**

(1) Background: The role of cytoreductive nephrectomy (CN) is controversial in patients with primary metastatic renal cell carcinoma (mRCC). (2) Methods: We evaluated the impact of CN, or no CN, followed by first-line targeted therapy (TT) in a nationwide unselected cohort of 437 consecutive patients with primary mRCC over a two-year period with a minimum of five years of follow-up. Data sources were national registries supplemented with manually extracted information from individual patient medical records. Cox proportional hazards estimated the hazard ratio (HR) of overall death and cancer-specific death after one and three years. (3) Results: 210 patients underwent CN and 227 did not. A total of 176 patients (40%) had CN followed by TT, 160 (37%) had TT alone, 34 (8%) underwent CN followed by observation, and 67 (15%) received no treatment. After adjustments in Model 2, patients treated with TT alone demonstrated a worsened overall survival (OS) compared to those treated with CN + TT, HR 0.63 (95% CI: 0.19–2.04). (4) Conclusions: In this nationwide study, CN was associated with enhanced outcomes in carefully selected patients with primary mRCC. Further randomized trials are warranted.

## 1. Background

Around 20% of patients with renal cell carcinoma (RCC) have primary metastatic disease, i.e., have a synchronous primary tumor and metastases at the time of diagnosis; the outcomes of these patients are poor [1,2,3]. The strategy of conducting cytoreductive nephrectomy (CN) followed by systemic therapy has been controversial [3,4,5]. In the cytokine era, two randomized trials demonstrated a modest survival improvement by combining upfront CN with interferon therapy, compared with interferon alone [2]. In the TKI era the pivotal CARMENA and SURTIME trials investigated the traditional approach of upfront CN followed by systemic therapy. CARMENA’s findings suggest that sunitinib alone might be superior to the combination of CN followed by sunitinib, challenging the necessity of CN in all mRCC cases. SURTIME explored the timing of CN, suggesting potential benefits of deferred CN after systemic therapy in certain patient subsets [6,7], and this finding has challenged the paradigm of CN [4].

We evaluated the impact of CN, or no CN, followed by first-line targeted therapy in a nationwide cohort of consecutive patients over a two-year period and with a minimum of five years of follow-up.

## 2. Methods

### 2.1. Patient Population

The target group in this study were RCC stage IV patients registered in the Danish Renal Cancer Database (DaRenCa) between 1 January 2014 and 31 December 2016, with a follow-up of a minimum of 5 years. Metastasis was defined as local or distant metastases evident at least 120 days within initial diagnosis. Data were collected from national registries supplemented with information extracted manually from individual patient medical records. Registries included DaRenCa [5], the Civil Registration System (CRS) [8], The National Patient Register (NPR) [9], The Danish Pathology Register (DPR) [10], The Danish Cancer Register (DCR) [11], and The Danish Causes of Death Register [12].

### 2.2. Exposure of Interest

In this study, symptoms present at the time of diagnosis, Body Mass Index (BMI), hypertension, surgical margin, primary metastatic disease, multidisciplinary team conference (MDT) involvement, time from initial diagnosis to metastasis, and treatment of metastatic RCC (mRCC) were exposures of interest for overall and cancer-specific death.

### 2.3. Research Variables

The following variables were obtained from patient medical records: age, gender, BMI, the presence of symptoms at the time of diagnosis, smoking status, hypertension and medication for hypertension, the Eastern Cooperative Oncology Group (ECOG) Performance status. Data were categorized as 0, 1–2, or 3–4. Categorization into International Metastatic Renal Cell Carcinoma Database Consortium (IMDC) risk groups was allocated.

### 2.4. Clinical Characteristics

Patients were characterized based on tumor histological subtypes (clear cell RCC (ccRCC) and non-clear cell RCC (non-ccRCC)) and T-stage (T-stage was assigned according to 2009 TNM classification) for analysis.

### 2.5. Treatment

Information about whether the patient had been discussed at an MDT conference before surgery was found in medical journals. The type of surgery was categorized as “open radical nephrectomy”, “laparoscopic radical nephrectomy”, “open partial nephrectomy”, “laparoscopic partial nephrectomy”, “ablation therapy”, or “no surgery”. The surgical margin was defined as positive when the tumor margin was not resected radically; coded in the analysis as “positive”, “negative”, or “no surgery”. Information about CN (yes/no) and lymphadenectomy (yes/no) was found in medical records or in the DPR.

Criteria for performing CN were based on the following criteria: CN was technically feasible based on CT assessment; the patient had a performance status of 0 or 1; the patient was clinically stable; there was no comorbidity of major clinical impact.

### 2.6. Outcomes

Events of interest were death (any cause or death due to RCC). Information about death was ascertained through the CRS and the Causes of Death Registry. Information about metastasis was obtained through medical records and computerized tomography (CT) scan reports.

### 2.7. Follow Up

Patients’ risk time was defined from diagnosis until an event of interest, or last follow-up until December 2021. Data collection was performed between May 2020 and December 2021. The Danish Patient Safety Authority granted permission to extract information from the medical records of patients involved in the study, as per Danish legislation (3-3013-2902/1), and the data were stored according to the Danish Data Protection Agency (REG-041-2021).

### 2.8. Statistical Methods

Analyses were performed separately for ccRCC and non-ccRCC. Descriptive statistics, including frequencies for categorical variables and median and interquartile range (IQR) for continuous variables, are presented in Table 1. Multiple imputations were used to impute missing values for all variables included in the models. Data were imputed 50 times and Rubin’s Rule was used to combine the results from the 50 imputed models. Kaplan–Meier curves were estimated for all categorical variables. Cox proportional hazards (PH) regression was used to estimate the hazard ratio (HR) of overall death and cancer-specific death after one year and three years. The adjustment was performed stepwise. First, univariate models were performed for all exposures, followed by model 1 with adjustment for clinical characteristics (age, gender, Leibovich score and sarcomatoid differentiation). In model 2, adjustment for health-related patient characteristics was added to the previous model (model 1 + adjusted for smoking, hypertension, performance status, and decision taken by MDT). Analysis of surgical margin as the main exposure and the type of surgery (radical vs. partial nephrectomy) were included in both models.

## 3. Results

### 3.1. Patient Characteristics and Outcomes

Data for 437 patients with primary mRCC were collected from the DaRenCa database between the 1st of January 2014 and the 31st of December 2016. Out of 437 patients, 176 (40%) underwent CN followed by targeted therapy (TT), 160 (37%) received TT alone, 34 (8%) patients had CN followed by observation, and 67 (15%) had no treatment (palliative therapy). For the 336 patients involved in our study, the TT agents employed across diverse treatment sequences were categorized as follows: Sunitinib: 83 cases, 24%, Pazopanib: 195 cases, 58%, Sorafenib: 3 cases, 0.8%, Temsirolimus: 11 cases, 3%, Interleukin-2/Interferon: 13 cases, 3.8%, Bevacizumab: 1 case, 0.2%, Atezolizumab/Bevacizumab: 16 cases, 4.5%, Pembrolizumab: 9 cases, 2.3%, Ipilimumab/Nivolumab: 5 cases, 1.4%, Nivolumab: 2 cases, 0.5%, Cabozantinib: 1 case, 0.2%, Axitinib: 2, cases, 0.5%, others: 3 cases, 0.8%.

The median time from CT to TT was 30 days (IQR: 21–44 days).

The study included 34% female patients and 66% male patients. For patients receiving CN alone, TT alone, CN + TT, or no therapy, the mean BMI was 25, 26, 26 and 25, respectively (Table 1). On average, pain and weight loss were the most prevailing symptoms. The most common histology was ccRCC (82%). The most frequent sites of metastasis were lungs, followed by bone, liver, and brain. The baseline characteristics of patients included in the study are shown in Table 1.

### 3.2. Results of Univariate and Multivariable Analysis

The median OS for all patients was 13.9 months. The median OS for patients with CN alone was 30.5, for patients with CN followed by TT 23.0 months, for patients with TT alone 11.9 months, and for patients without treatment 2.8 months. Based on the IMDC score levels, the median OS for patients with favorable/intermediate scores was 21.2 months and for patients with a poor score was 9.7 months.

Multivariable analysis showed patients undergoing CN + TT had improved OS with HR 0.66 (95% CI: 0.34–1.28), compared to TT only (HR 1.24), and no-treatment (HR 4.71), with CN-only followed by observation as a reference (Supplementary Table S1).

After multivariable adjustment in the final model 2, and excluding patients that did not receive any treatment, patients who received TT had poorer OS HR 2.57 (1.44–4.58) than patients who underwent CN plus TT HR 1.62 (0.90–2.93) (Table 2). The number of sites with metastasis (liver, brain, or bone) did not reach statistical significance: 1 site, 0 sites HR 0.82 (0.62–1.09), 2 sites HR 0.86 (0.56–1.30), 3 sites HR 2.08 (0.32–13.66) (Table 2). Mortality was only affected by the IMDC classification, where patients in the intermediate/favorable risk group compared to the poor risk group had significantly improved survival; HR 0.56 (0.41–0.77) (Table 2).

**Figure 1:**
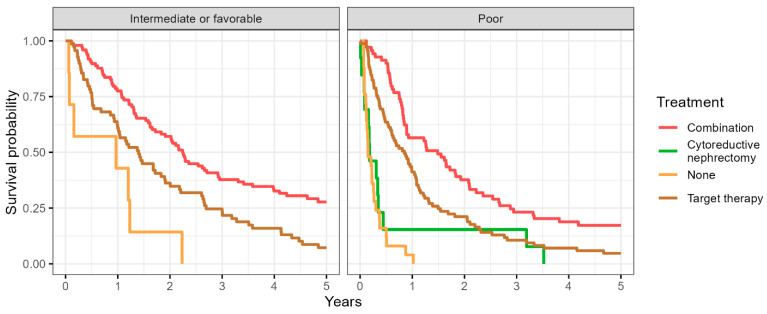
Representing the Kaplan–Meier curve showing the overall survival probability in patients receiving different treatments accounting for the IMDC risk in the final analysis.

The overall survival probability was improved in patients who received CN followed by TT to those who received CN alone, TT alone or no treatment; this was apparent in both the IMDC favorable/intermediate risk group and the IMDC poor risk group (Figure 1).

## 4. Discussion

The present study, a nationwide consecutive cohort of patients, showed that patients with synchronous mRCC undergoing CN plus TT had improved survival. Criteria for performing CN were discussed at an MDT conference and based on the following criteria: CN was technically feasible based on CT assessment; the patient had a performance status of 0 or 1; the patient was clinically stable; there was no comorbidity of major clinical impact.

Improved OS with CN + TT was seen irrespective of the presence of brain, liver, or bone metastases, and irrespective of IMDC risk classification, despite IMDC risk being the only factor associated with impaired OS in our analysis. Thus, CN may still serve as an effective surgical intervention in carefully selected patients.

This study’s findings reveal a noteworthy trend in patient outcomes based on the treatment modalities employed. Patients who were treated exclusively with TT exhibited a reduction in OS when compared to those who underwent a combination of CN and TT. It is important to note, however, that this observed difference in survival rates did not reach statistical significance. This suggests that other factors, potentially external to the treatment modalities themselves, might have influenced these outcomes. A critical aspect that merits consideration is the varying degrees of disease progression among the patient groups. Specifically, the group receiving only TT might have had more advanced stages of the disease, which could contribute to their lower survival rates. This observation underscores the vital importance of patient selection in clinical treatment planning. It also highlights the indispensable role of an MDT in the treatment of such complex cases.

The CARMENA trial [6] included less than one patient per year per participation center; therefore, despite being a randomized trial, the results are subject to selection bias. This study presents real-world data from unselected patients and demonstrates a statistically significant and clinically relevant improved OS in all IMDC risk groups, irrespective of metastatic location, employing simple and strict clinical selection criteria utilized in the MDT setting. However, we acknowledge the weaker retrospective design in our study.

For 20 years, there have been signs trending towards CN benefiting patients in terms of improved overall survival in patients with primary mRCC. This was reported in the combined analysis of CN plus interferon compared with interferon alone in the cytokine era [13]. In the targeted therapy era, repeated notions regarding the positive association between CN and improved survival have been published, both in ccRCC as well as in non-cc RCC [14,15,16,17,18,19,20,21,22]; our data are in agreement with these findings. Additionally, in the checkpoint immunotherapy era, the potential benefits of performing CN in patients with primary mRCC have been reported. Importantly, a lower objective response rate, especially a lower complete response rate, as well as a shorter OS in patients without CN, was noted in the post hoc analysis in patients with an evaluable primary renal tumor in the CheckMate 214 trial [23]. Furthermore, there is a consensus that synchronous mRCC (i.e., primary metastatic RCC) compared with metachronous mRCC is associated with poorer outcomes following systemic therapy [3]. Thus, our data in an unselected nationwide consecutive cohort of patients supported CN being considered as part of the multidisciplinary agenda. Within recent years, the treatment paradigm has shifted towards IO-IO or a TKI-IO combination treatment for all patients with primary metastatic RCC [24]. This necessitates new studies evaluating the effect of CN in primary metastatic RCC patients treated with contemporary IO-based therapy. There are currently two ongoing trials, the NORDIC-SUN trial (NCT03977571) and the PROBE trial (NCT04510597). Both studies are evaluating the deferred approach of CN in cohorts of primary metastatic RCC treated with IO-based combinations. Furthermore, the NORDIC-SUN trial carries a comprehensive translational research program with microbiome, tissue and blood sampling for biomarker analysis and future personalized management [25].

The strengths of the study include it being a multicenter study with the inclusion of stage IV patients in Denmark between 2014 and 2016. Due to the use of registry data, we had full follow-up on all patients included. Use of registries and extraction from medical records enabled data on multiple key variables. Application of multiple imputations with a high number of imputations allowed efficient use of the cohort. Limitations were the retrospective design, no adjustment for socioeconomic position, and comparatively fewer patients with non-ccRCC, resulting in a risk of over-adjustment. Further, a new immune treatment was introduced in 2015, which may bias results, even though a rather small proportion of the study population received this treatment. Another limitation to be addressed is the potential for selection bias and misclassification in the CN-alone group. It is plausible that some patients initially categorized as having metastatic lesions may have actually had non-metastatic renal cell carcinoma (RCC). This potential bias will emphasize the necessity for cautious interpretation of the results within the CN-alone group.

## 5. Conclusions

In this nationwide study of patients with primary mRCC, CN was associated with improved outcomes for carefully selected patients. Further randomized trials are warranted.

## Figures and Tables

**Table 1:** Patients’ baseline clinical characteristics undergoing metastatic renal cancer.

Characteristics	N = 437	CN Alone	TT Alone	CN + TT	No-Treatment	*p* Value
		34 (7.78%)	160 (36.61%)	176 (40.27%)	67 (15.33%)	
Age, median (IQR)	66 (14)	68 (13)	66 (12)	64 (14)	71 (13)	<0.001
Female gender, n (%)	147 (34%)	11 (32%)	55 (34%)	60 (34%)	21 (31%)	0.971
BMI, median (IQR)	25 (6)	25 (5)	26(7)	25 (5)	24(5)	0.793
NA *	30	<5	12	<5	14	
Symptoms, n (%)						
Hematuria	90 (21%)	11 (28%)	30 (18%)	41 (25%)	8 (13%)	0.070
Pain	115 (27%)	14 (36%)	41 (25%)	43 (27%)	17 (27%)	0.234
Weight loss	129 (30%)	7 (18%)	52 (32%)	47 (29%)	23 (37%)	0.334
Other	92 (22%)	7 (18%)	40 (25%)	30 (19%)	15 (23%)	0.351
Smoking, n (%)						
Currently	125 (29%)	10 (29%)	41 (26%)	49 (28%)	25 (37%)	0.738
Previously	160 (37%)	12 (35%)	59 (37%)	69 (39%)	20 (30%)	
Never	126 (29%)	<15	41 (26%)	<65	<25	
NA *	26 (6%)	<5	19 (12%)	<5	<5	
Hypertension						
Yes	243 (56%)	19 (56%)	85 (53%)	94 (53%)	45 (67%)	0.188
No	183 (42%)	<20	<75	77 (44%)	<25	
NA *	11 (3%)	<5	<5	5 (3%)	<5	
ASA Score, n (%)						0.014
1	49 (11%)	<5	19 (12%)	26 (15%)	<5	
2	224 (51%)	20 (59%)	75 (47%)	103 (59%)	26 (39%)	
≥3	117 (27%)	10 (29%)	39 (24%)	41 (23%)	27 (40%)	
NA *	47 (11%)	<5	27	6 (3%)	<15	
Performance Status, n (%)						
0	152 (35%)	11 (32%)	45 (28%)	91 (52%)	5 (7%)	<0.001
1	178 (41%)	13 (38%)	76 (48%)	73 (41%)	16 (24%)	
≥2	<105	<10	<40	<15	<45	
NA *	<10	<5	<5	<5	<5	
Decision taken in MDT, n (%)	290 (66%)	21 (62%)	111 (69%)	122 (69%)	36 (54%)	0.092
Subtype of RCC						
ccRCC	357 (82%)	25 (74%)	112 (70%)	165 (94%)	55 (82%)	<0.001
Non-ccRCC	<80	<10	<50	<15	<15	
NA *	<5	<5	<5	<5	<5	
T-stage, n (%)						0.027
Tx						
T1	64 (15%)	5 (15%)	30 (19%)	16 (9%)	13 (19%)	
T2	49 (11%)	<5	16 (10%)	<30	5 (7%)	
T3	230 (53%)	20 (59%)	67 (42%)	108 (61%)	35 (52%)	
T4	66 (15%)	6 (18%)	28 (18%)	24 (14%)	8 (12%)	
NA *	28 (6%)	<5	19 (12%)	<5	6 (9%)	
Fuhrman grade, n (%)						
Non-ccRCC	78 (18%)	8 (24%)	47 (29%)	11 (6%)	12 (18%)	
I–II	78 (18%)	<10	30 (19%)	<30	15 (22%)	<0.001
III–IV	220 (50%)	20 (59%)	42 (26%)	135 (77%)	23 (34%)	
NA *	61 (14%)	<5	41 (26%)	<5	17 (25%)	
Necrosis, n (%)	247 (57%)	21 (62%)	55 (34%)	148 (84%)	23 (34%)	<0.001
N stage, n (%)						
Nx						
N0	258 (59%)	24 (71%)	83 (52%)	114 (65%)	37 (55%)	0.045
N1	179 (41%)	10 (29%)	77 (48%)	62 (35%)	30 (45%)	
IMDC, n (%)						<0.001
Favorable	9 (2%)	<5	<5	7 (4%)	<5	
Intermediate	165 (38%)	<5	<70	91 (52%)	<10	
Poor	192 (44%)	13 (38%)	85 (53%)	69 (39%)	25 (37%)	
NA *	71 (16%)	21 (62%)	6 (4%)	9 (5%)	35 (52%)	
Sarcomatoid, n (%)	86 (20%)	14 (41%)	18 (11%)	49 (28%)	5 (7%)	<0.001
Tumor size, mean (SD)	90 (39)	89 (41)	86 (42)	95 (37)	85 (38)	0.025
NA *	26	<5	16	<5	8	
Type of surgery, n (%)						
None	190 (43%)	<5	<135	<5	<60	<0.001
Laparoscopy	110 (25%)	13 (38%)	10 (6%)	81 (46%)	6 (9%)	
Open	127 (29%)	19 (56%)	14 (9%)	89 (51%)	5 (7%)	
NA *	10 (2%)	<5	<5	<10	<5	
Metastasis sites, n (%)						
Lung	293 (67%)	19 (56%)	99 (62%)	132 (75%)	43 (64%)	0.027
Liver	85 (19%)	7 (21%)	34 (21%)	32 (18%)	12 (18%)	0.887
Bone	187 (43%)	10 (29%)	80 (50%)	72 (41%)	25 (37%)	0.073
Brain	43 (10%)	<5	13 (8%)	24 (14%)	<5	0.163
Other	173 (40%)	13 (38%)	64 (40%)	71 (40%)	25 (37%)	0.974

* NA = missing data.

**Table 2:** Multivariable adjusted 3-year mortality hazard ratios (HRs) with corresponding 95% confidence intervals (CIs) for risk of death among patients diagnosed with metastatic renal cancer in Denmark 2014–2016.

Exposures	MODEL 1 ^A^	MODEL 2 ^B^
	HR (95% CI)	HR (95% CI)
Age		
Age ≤ 70	1	1
Age > 70	1.05 (0.81–1.35)	1.01 (0.77–1.33)
Treatment		
CN only	1	1
TT only	2.66 (1.50–4.72)	2.57 (1.44–4.58)
CN plus TT	1.72 (0.96–3.08)	1.62 (0.90–2.93)
No treatment	5.96 (3.10–11.45)	5.94 (3.06–11.52)
IMDC		
Poor	1	1
Intermediate or favorable	0.56 (0.42–0.76)	0.56 (0.41–0.77)
Symptoms		
No	1	1
Yes	0.99 (0.76–1.29)	0.97 (0.73–1.28)
Tumor size		
Tumor < 100 mm	1	1
Tumor ≥ 100 mm	1.35 (1.05–1.75)	1.35 (1.04–1.76)
Gender		
Male	1	1
Female	1.14 (0.89–1.46)	1.15 (0.89–1.48)
Liver + brain + bone sites		
1	1	1
2	0.87 (0.58–1.31)	0.86 (0.56–1.30)
3	2.10 (0.34–13.05)	2.08 (0.32–13.66)
0	0.82 (0.62–1.08)	0.82 (0.62–1.09)

^A^ Model 1: Adjusted for age, gender, tumor stage, tumor size, Fuhrman grade, necrosis status and sarcomatoid. ^B^ Model 2: Model 1 + adjusted for BMI, symptoms, smoking, hypertension, ASA score and the decision made in MDT.

## Data Availability

All data relevant to this study are contained within the manuscript. No additional data beyond that presented in the manuscript can be provided.

## Supplementary Materials

The following supporting information can be downloaded at: https://www.mdpi.com/article/10.3390/cancers16061132/s1, Table S1: Multivariable adjusted 1-year mortality hazard ratios (HRs) with corresponding 95% confidence intervals (CIs) for risk of death among patients diagnosed with metastatic renal cancer in Denmark 2014–2016.

## Data Protection

All data were stored and managed in compliance with the guidelines and requirements of the Danish Data Protection Agency (Registration Number: REG-041-2021).
