# Supplementary material for: Cytoreductive Nephrectomy in Select Primary Metastatic Renal Cell Carcinoma Patients: A Comprehensive Nationwide Outcome Analysis
Source: Cancers (Basel). 2024 Mar 12;16(6):1132. doi: 10.3390/cancers16061132 (PMC10968994; doi:10.3390/cancers16061132)
Supplement: Supplementary file 1 [file cancers-16-01132-s001.zip › cancers-2811463-supplementary.pdf]

Supplementary Table S1: Multivariable adjusted 1-year mortality hazard ratios (HRs) with corresponding 95% confidence intervals (CIs) for risk of death among patients diagnosed with metastatic renal cancer in Denmark 2014-2016.

Exposures	Model 1a<br>HR (95% CI)	MODEL 2B<br>HR (95% CI)
Age		
Age <=70	1	1
Age >70	0.99 (0.71-1.38)	1.01 (0.70-1.45)
Treatment		
CN only	1	1
TT only	1.35 (0.72-2.55)	1.24 (0.65-2.38)
CN plus TT	0.74 (0.39-1.41)	0.66 (0.34-1.28)
No treatment	4.58 (2.26-9.28)	4.71 (2.28-9.73)
IMDC		
Poor risk group	1	1
Favorable and Intermediate risk group	0.47 (0.32-0.70)	0.46 (0.30-0.70)
Symptoms		
No	1	1
Yes	1.02 (0.71-1.46)	1.03 (0.71-1.50)
Tumor size		
Tumor <100 mm	1	1
Tumor >=100mm	1.55 (1.12-2.16)	1.59 (1.13-2.24)
Gender		
Male	1	1
Female	1.10 (0.79-1.53)	1.12 (0.80-1.57)
Liver+brain+bone sites		
1	1	1
2	0.95 (0.55-1.63)	0.87 (0.50-1.54)
3	1.91 (0.17-21.44)	2.30 (0.18-29.48)
0	0.95 (0.67-1.36)	0.93 (0.65-1.34)

AModel 1: Adjusted for age, gender, tumor stage, tumor size, Fuhrman grade, necrosis status, sarcomatoid
BModel 2: Model 1 + adjusted for BMI, symptoms, smoking, hypertension, ASA score, MDT decision
